# Behavior of osteoblastic cells cultured on titanium and structured zirconia surfaces

**DOI:** 10.1186/1746-160X-4-29

**Published:** 2008-12-08

**Authors:** Rita Depprich, Michelle Ommerborn, Holger Zipprich, Christian Naujoks, Jörg Handschel, Hans-Peter Wiesmann, Norbert R Kübler, Ulrich Meyer

**Affiliations:** 1 Department of Cranio- and Maxillofacial Surgery, Heinrich-Heine-University, Düsseldorf, Germany; 2 Department of Operative and Preventive Dentistry and Endodontics, Heinrich-Heine-University, Düsseldorf, Germany; 3 Department of Prosthetic Dentistry, Section of Materials Sciences, Johann Wolfgang Goethe University, Frankfurt, Germany; 4 Department of Cranio- and Maxillofacial Surgery, Westphalian Wilhelms-University, Münster, Germany

## Abstract

**Background:**

Osseointegration is crucial for the long-term success of dental implants and depends on the tissue reaction at the tissue-implant interface. Mechanical properties and biocompatibility make zirconia a suitable material for dental implants, although surface processings are still problematic. The aim of the present study was to compare osteoblast behavior on structured zirconia and titanium surfaces under standardized conditions.

**Methods:**

The surface characteristics were determined by scanning electron microscopy (SEM). In primary bovine osteoblasts attachment kinetics, proliferation rate and synthesis of bone-associated proteins were tested on different surfaces.

**Results:**

The results demonstrated that the proliferation rate of cells was significantly higher on zirconia surfaces than on titanium surfaces (p < 0.05; Student's *t*-test). In contrast, attachment and adhesion strength of the primary cells was significant higher on titanium surfaces (p < 0.05; *U *test). No significant differences were found in the synthesis of bone-specific proteins. Ultrastructural analysis revealed phenotypic features of osteoblast-like cells on both zirconia and titanium surfaces.

**Conclusion:**

The study demonstrates distinct effects of the surface composition on osteoblasts in culture. Zirconia improves cell proliferation significantly during the first days of culture, but it does not improve attachment and adhesion strength. Both materials do not differ with respect to protein synthesis or ultrastructural appearance of osteoblasts. Zirconium oxide may therefore be a suitable material for dental implants.

## Background

The objective of implantology is to design devices that induce controlled, guided, and rapid integration into surrounding tissues [1]. Events leading to integration of an implant, and ultimately to success or failure of the device, take place largely at the tissue-implant interface, and osteoblasts covering the implant surface are the crucial cell type that regulate the tissue response at the biomaterial surface [2]. Based on the results of numerous *in vitro *studies, it is now well understood that surface morphology decisively determines the cellular behavior of osteoblasts [2-4].

Titanium (Ti) and titanium alloys are widely used as implant materials due to their excellent biocompatibility. Many surface modifications have been developed to improve cell reactions on the surface. In addition to existing titanium implants bearing machined or plasma-sprayed surfaces, there is a great number of implants on the market which offer surfaces altererd by grit blasting and/or acid etching. Zirconia (zirconium dioxide, ZrO_2_) is a bio-inert non-resorbable metal oxide that offers improved mechanical properties compared to other ceramic biomaterials, i.e. alumina. It has a good chemical and dimensional stability, and a high strength and toughness [5]. Tetragonal zirconia polycrystals (TZP) are used for manufacturing femoral heads for total hip replacements since the late 1980s [6]. Because of the tooth-like colour, the excellent biocompatibility and mechanical properties, ambitious efforts were made to introduce zirconia for applications in dentistry. Successful use of zirconia for treatment of non-vital teeth [7,8], crown and bridge restorations [9] and ceramic abutments [10] are reported. Zirconia is also a desirable alternative material to titanium for the fabrication of dental implants.

Titanium has a superior corrosion resistance because of its characteristic oxide layer, however, accumulation of titanium in the inner organs and lymph nodes after implantation has been reported [11]. Galvanic side effects after contact with saliva and fluoride were also described [12]. Although allergic reactions to titanium are very rare, cellular sensitization has been demonstrated [13,14]. The main disadvantage of the biomaterial titanium is its dark grayish colour. Unfavorable soft tissue conditions or retraction of the gingiva may lead to aesthetic impairment, especially when the maxillary incisors are involved [15]. The clinical use of zirconia is limited, because fabrication of surface modifications is difficult and smooth implant surfaces are not beneficial for osseointegration, due to a poor interaction with tissues [1].

Some animal experiments and numerous case reports demonstrated osseointegration of zirconia implants similar to that of titanium implants, suggesting that zirconia might be a suitable implant material [16-19]. However, data evaluating the role of surface topography on the response of osteoblasts at zirconia interfaces are rare [20]. Cell reactions on surfaces are strongly dependent on the culture system that is used [21]. Since most of the widely used osteosarcoma cell lines do not demonstrate a complete pattern of osteoblastic features *in vitro*, the use of primary non-transformed cells seems to be superior for assessing of osteoblast reactions on biomaterial surfaces [2]. Therefore, the aim of this study was to compare osteoblast behavior on structured zirconia and titanium surfaces under standardized conditions using primary bovine osteoblasts. Attachment kinetics, proliferation rate, and synthesis of bone-associated proteins on both surfaces were examined and compared between each other.

## Methods

A modified (acid-etched) zirconia implant surface was compared to an acid-etched titanium surface. Standard 24-well tissue culture plates (polystyrene) were used as control surface. Zircona disks (12 mm diameter, 1 mm thick) were made of yttrium-stabilized tetragonal polycrystals and titanium disks (13 mm diameter, 1.5 mm thick) were made of commercially pure titanium. Both materials were supplied by Konus Dental Implants (Bingen, Germany). To evaluate the surfaces of zirconia and titanium disks, scanning electron microscopy (SEM) was performed using a a JEOL 6300F (JEOL, Eching, Germany) high-resolution field emission scanning electron microscope equipped with a EDX analysis system. The zirconia and titanium disks were carefully washed in diluted water, rinsed thoroughly in 70% ethanol, and ultrasonically cleaned for 20 min in absolute alcohol. Finally, the samples were air dried and maintained under sterile conditions after gamma ray sterilization.

### Primary osteoblast cell culture

Primary bovine osteoblasts were used in this study. Extraction and cultivation were performed following the instructions of Jones et al. [22]. Under sterile conditions periosteum was removed from the bovine metacarpus. The periosteum was cultured at 37°C in an atmosphere of 5% CO_2 _and 100% humidity for 4–5 weeks in high-growth enhancement medium (High GEM, Flow Laboratories, Rickmansworth, UK) containing 10% fetal bovine serum (FBS, Gibco Laboratories Grand Island, NY, USA). Media were changed weekly. Osteoblastic differentiation was tested by detection of osteocalcin/osteonectin and high alkaline phosphatase activity. When the cells reached confluence they were harvested (20 min incubation at 37°C with 0.4 g collagenase, 98.8 mg HAM's F10 in 10 ml HEPES (2-[4-(2-hydroxyethyl)-1-piperazinyl]ethanesulfonic acid); repeated washing with phosphate-buffered saline (PBS); subsequent incubation for 15 min with 300 mg ethylenediaminetetraacetic acid (EDTA)-Na, 200 mg KCl, 8 g NaCl, 1 g NaHCO_3_, 50 mg NaH_2_PO_4 _and 1 g glucose/l) and centrifuged. The pellets were resuspended with buffer and the cell numbers were counted in a cell counter (CASY^®^I Modell TT, Schärfe System, Reutlingen, Germany).

### Cell proliferation

Cell proliferation was measured after 1, 3 and 5 days, respectively. Cells were marked with fluorescent dye (Vybrant^® ^CM DiI, Molecular Probes, Netherlands) and 10.000/cm^2 ^osteoblasts were seeded into 24-well plates on the zirconia/titanium disks or the well plate. The experiments were repeated at least three times. Osteoblasts were fixed in methanol and stained with methylene blue and azure blue according to the method described by Richardson. Morphometric evaluation of cells was performed by means of light microscopy. To determine the cell number digital photos were taken under standardized conditions and counted using the software program Analysis 3.0 (Olympus Soft Imaging System, Münster, Germany).

### Cell detachment

To determine cell adhesion on the surface of the different materials, 60.000/cm^2 ^primary osteoblasts were seeded into 24-well plates on the zirconia/titanium disks or the well plate. After incubation for 24 hrs at 37°C, 500 μl of a trypsin-containing solution (0.25% diluted 1:2 in PBS) was added and 400 μl aliquots of the cell suspension were taken after a contact time of 5, 15, 25, and 35 min. Cell numbers were determined by the use of a cell counter. As control, the remaining of the 500 μl was removed from the wells and 500 μl trypsin (0.25% solution, non-diluted) was added to detach the remaining cells. After 5 min contact time and washing with PBS, aliquots of the cell suspension (400 μl) were taken and the cell number counted.

### Immunocytochemistry

To test for osteoblastic differentiation, expression of collagen I, osteocalcin and osteonectin was assessed by means of immunocytochemistry. 60.000 osteoblasts/cm^2 ^were seeded into 24-well plates on the zirconia/titanium disks and into 6-well plates on polystytol. After incubation for 7, 14, or 28 days at 37°C in an atmosphere of 5% CO_2 _in the High GEM medium, primary antibodies were used according to the manufacturers' instructions: rabbit polyclonal anti-collagen I (Biotrend, Cologne, Germany), Mouse monoclonal anti-osteocalcin (TaKaRa Bio, MoBiTec, Goettingen, Germany) and rabbit polyclonal anti-osteonectin (SPARC; Chemicon Millipore GmbH, Schwalbach, Germany). Alexa Flour 488-labelled secondary antibodies were purchased from MoBiTec (Goettingen, Germany) and used according to the manufacturers' instructions. Digital images were taken under standardized conditions using a fluorescence microscope and processed using the software program Analysis 3.0.

### Scanning electron microscopy (SEM)

Cell morphology was investigated after 2 hrs, 4 hrs and 7 days. Primary osteoblasts were seeded at a density of 15.000/cm^2 ^on zirconia/titanium disks and for control on smooth titanium disks and incubated for 2 hrs or 4 hrs at 37°C in an atmosphere of 5% CO_2 _in the High GEM medium. To investigate confluent cells after 7 days, 40.000/cm^2 ^osteoblasts were seeded on the zirconia/titanium disks and incubated under the same conditions. Cells were fixed in 2.5% glutaraldehyde for 3 hrs and then washed with PBS. After sputtering with gold (Bal-tec Ag, Balzers, Liechtenstein) the samples were investigated using the scanning electron microscope JEOL 6300F (JEOL, Eching, Germany).

### Statistical analysis

Statistical analyses were performed using Student's *t*-tests and Mann-Whitney *U *tests. A p < 0.05 was considered significant. Experiments were repeated three-fold.

## Results

### Surface topography

**Figure 1:**
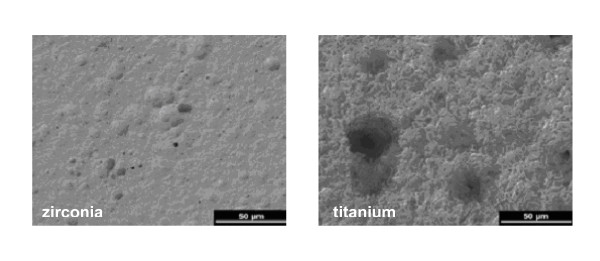
Scanning electron micrographs of a zirconia disk (left) showing occasionally pores on the smooth surface and a titanium disk (right) with rough surface and frequent pores and grooves of different size (2 kV, magnification 500-fold).

Scanning electron microscopy demonstrated noticeable differences between zirconia and titanium surfaces by SEM revealed (Figure 1). The titanium surface was rough and contained many pores and grooves of different size which were regularly distributed over the whole surface. In contrast, the zirconia surface appeared smooth with only a few pores.

### Energy-dispersion X-ray analysis

Energy-dispersion X-ray analysis confirmed the characteristic element composition of commercial pure titanium and zirconium dioxide. Titanium disks were composed of the elements titanium and oxygen but also traces of silicium and carbon were detected. Zirconia consisted of zirconium (Zr) and oxygen (O), but also hafnium (Hf) was found frequently associated with ZrO_2_.

### Cell proliferation

**Figure 2:**
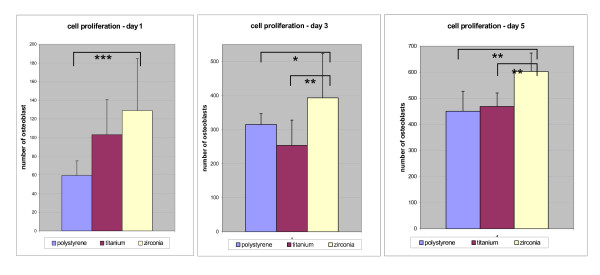
Cell proliferation rates of osteoblasts on differently coated surfaces at day 1, 3 and 5, respectively. Increase in cell number was detected on all surfaces over the observation period. Significantly higher cell proliferation was observed on zirconia surfaces on day 1, 3 and 5 compared to titanium and polystyrene surfaces. Statistical differences (p < 0.05) as calculated by Student's *t*-tests are marked with arrows.

Cell proliferation was assessed on the different surfaces. We found an increase in cell number on all surfaces over the observation period (Figure 2). At day 1 cell proliferation was significantly higher on zirconia surfaces as compared to polystyrene controll surfaces (p = 0.000) but was similar to titanium surfaces (p = 0.158). At day 3 cell growth was significantly higher on the zirconia surfaces than on polystyrene (p = 0.037) and titanium surfaces (p = 0.002). At day 5 cell proliferation was continued to be significantly higher on zirconia surfaces than on titanium (p = 0.001) or polystyrene surfaces (p = 0.001).

### Cell detachment

Results revealed that at every time of the assessment fewer cells were detached from titanium surfaces compared to zirconia or polystyrol surfaces. The number of detached cells from titanium surfaces remained constant at a low level over the whole period of investigation. In contrast, detached cells from zirconia surfaces doubled from 5 to 15 min, but remained constant thereafter. A minor increase of detached cells was found in the polystyrol control group, and after 35 min the number of detached cells had quadrupled. Statistical analysis confirmed significant higher cell detachment rates from zirconia surfaces as compared to titanium surfaces after 5 min (p = 0.047), 15 min (p = 0.009) and 25 min (p = 0.009) but not after 35 min (p = 0.1). Differences between zirconia and control group were not significant (p < 0.05) at any time of assessment.

### Immunocytochemical analysis

**Figure 3:**
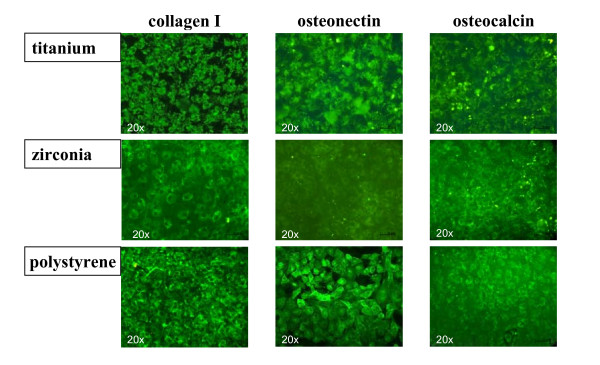
Immunocytochemical analysis of characteristic bone derived proteins. After 7 days extracellular expression of collagen I and osteonectin is evident on all different surfaces examined. Scattered expression of osteocalcin is demonstrated (magnification 20-fold).

**Figure 4:**
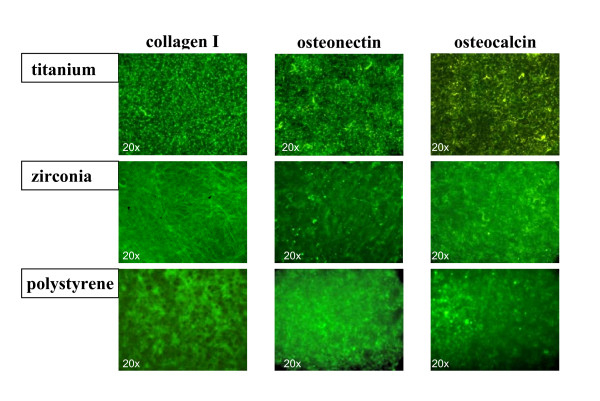
After 28 days expression of collagen I, osteocalcin and osteonectin is still evident on all different surfaces examined. Minimally denser accumulation of reticular collagen fibrils on zirconia surfaces as compared to titanium surfaces are observed (magnification 20-fold).

After 7 days expression of collagen I, osteocalcin and osteonectin were evident on all different surfaces examined. Cells were uniformly distributed throughout the material surface and positive immunolabeling was detected on zirconia, titanium and polystyrol surfaces. Lower expression of osteocalcin compared to collagen I and osteonectin was observed on all different surfaces (Figure 3). After 14 days of culture, up-regulated expression of reticular collagen I expression was evident especially on the titanium and zirconia surfaces, whereas osteocalcin and osteonectin expression showed no detectable differences on the investigated surfaces. Expression of characteristic bone derived proteins was still detectable after 28 days on all samples and showed no significant differences between titanium, zirconia and polystyrol surfaces except of a minimally denser accumulation of collagen I found on zirconia surfaces as compared to titanium surfaces (Figure 4).

### Scanning electron microscopy (SEM)

**Figure 5:**
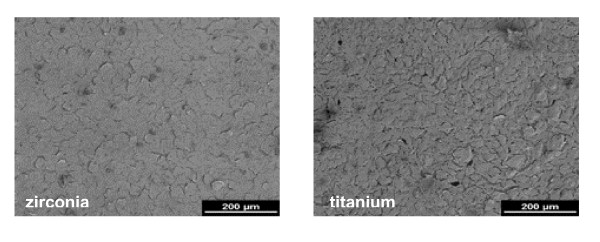
Osteoblasts after 7 days incubation showing a dense confluent cell layer on both zircona (left) and titanium surfaces (2 kV, magnification 100-fold).

The SEM analysis performed on osteoblast-seeded samples after 2 hrs showed typically flat polygonal cells regularly distributed on the titanium and on the zirconia surfaces. Development of radiate cell filopodia was apparent. After 4 hrs of culture, cell morphology on both surfaces showed no significant differences and was similar to that after 2 hrs. Cell filopodia exploring the surface could be demonstrated in fixed cells. After 7 days a mosaic-shaped confluent cell layer had formed on zircona and titanium surfaces (Figure 5). No ultrastructural signs of apoptotic fibroblast-shaped cells were detected. Significant differences could not be found.

## Discussion

Substratum composition and microtopography are important factors influencing growth and differentiation of osteoblasts [23]. The results of this study confirm previous observations that osteoblast-like cells react sensitive to surface roughness and material composition [24,25].

It was shown that osteoblast-like cells (MG63) grown on rough (titanium) surfaces exhibited reduced cell proliferation rate but increased alkaline phosphatase-specific activity and osteocalcin production [23,26,27]. In this study primary bovine osteoblasts were used as a culture model, because most transformed osteosarcoma cell lines do not demonstrate a complete pattern of *in vitro *differentiation. Substrate-dependent cell reactions are generally difficult to assess in cells derived from the osteoblastic lineage. Until now no study showed the reactions of primary osteoblasts on modified zircona surfaces and only a few studies focussed on cellular reactions of different osteoblast-like cells on zircona implant materials. Aldini et al. analysed *in vitro *and *in vivo *the reactions of osteoblast-like cells on zirconia surfaces that were either uncoated or coated with biological glass. Viability and metabolism of human osteoblast-like cells (HOS/TE85) were not affected by the presence of material extract in the culture [28]. Ko et al. also used HOS cells to investigate the initial bone cell response to pure titanium and zirconia/alumina composite ceramics ((Y, Nb)-TZP/alumina) and detected high cell proliferation rates and alkaline phosphatase activity at day 8. However expression of osteonectin showed no differences between titanium and ceramic materials [29]. Recently published studies analysed reactions of osteoblast-like cells (MG63) on zirconia surfaces using microarray techniques [30-32].

A specific pattern of differently regulated genes was detected. Bächle et al. [33]compared the growth of osteoblast-like osteosarcoma cells (CAL 72) on zirconia ceramics with different surface modifications to SLA titanium surfaces. After 3 days significantly lower proliferation rates were detected on the machined zirconia surface. After 6 and 12 days these differences were no longer detectable. After 12 days fully cell-covered areas were less frequently found on airborne particle-abraded and acid-etched zirconia surfaces, while high cell growth rates were observed on polystyrene surfaces. The authors concluded that cell morphology and cell-covered surface area were not affected by the type of substrate and that roughened zirconia is an appropriate substrate for the proliferation and spreading of osteoblastic cells.

Recently Rothamel and coworkers [19] investigated the biocompatibility and osseointegration of structured zirconia implants *in vitro *and *in vivo*. The growth of osteoblast-like SAOS-2 cells was significantly better on the machined zirconia surfaces compared to sand-blasted zirconia and polished titanium surfaces. The authors emphazised that manufacturing and cleaning processes may have an impact on the biocompatibilty of rough zirconia surfaces. Hoffmann et al. [34] observed a high degree of bone apposition on zirconia and titanium implants with comparable results for the two tested materials in a histologic evaluation in rabbits.

The results of our study showed cell growth and expression of characteristic bone proteins on all investigated surfaces. SEM observations demonstrated appropriate adhesion and spreading of cells on both zirconia and titanium surfaces. These results implicate a high biocompatibility of the used zirconia material. According to previous observations [25,35,36], cell proliferation rates were higher on smoother zirconia surfaces than on rougher titanium surfaces, suggesting that rough surfaces have no benefical effect on primary osteoblasts. This observation is in contrast to the widely used osteosarcoma cell lines MG 63 [3,27,36].

Ponader et al. [35] reported on higher growth rates of primary osteoblasts on compact smooth as compared to rough textured titanium surfaces but did not find effects of surface roughness on expression of osteogenic genes. According to these results, no different expression of osteoblast proteins on the zirconia or titanium surfaces was observed in this study. Fillies et al. [25] demonstrated increased synthesis of bone-specific matrix proteins, while other studies showed reduced alkaline phosphatase-specific activity in primary osteoblasts on rough surfaces [36]. Guizzardi et al. [37] detected no influence of surface topography on expression of characteristic osteoblast proteins. These controversial results underscore the complexity of osteoblast reactions on surface composition and topography. Hao et al. showed that an increased surface energy of magnesia-partially stabilized zirconia (MgO-PSZ) bioceramic after CO_2 _laser treatment resulted in higher initial cell attachment and enhanced cell growth of human foetal osteoblast cells (hFOB) [21,38].

In contrast to other authors [25,36], in the presented study increased cell attachment was detected on rough titanium surfaces as compared to smoother zirconia surfaces. Molecules involved in cell adhesion include extracellular matrix proteins, transmembrane receptors, and intracellular cytoskeletal components [33]. Zirconia ceramics are assumed to promote less intensive protein adsorption as compared to titanium and, in particular, polystyrene, and protein adsorption is a crucial factor for the initial cell adhesion on artificial surfaces [19]. The high cell detachment from the zirconia surfaces could also be due to the surface topography, because the zirconia surfaces showed less pores and irregularities than the titanium surfaces and osteoblasts prefer attaching into deep lying areas [35]. Further studies need to be conducted to investigate the complexity of osteoblast reactions on surface composition and topography of zirconia ceramics.

## Conclusion

The present study showed that primary bovine osteoblasts are able to attach, proliferate and differentiate on modified zirconia surfaces *in vitro*, suggesting that the ceramic material may also have beneficial effects on biocompartibility and osseointegration when used in patients.

## Competing interests

The authors declare that they have no competing interests.

## Authors' contributions

RD suggested the original idea for the study, supervised the study and did the statistical analysis, interpreted the data, reviewed and contributed to the writing of all iterations of the paper, including the final version of the manuscript. MO, CN, JH, HPW, UM participated in discussions on the undertaking of the study, interpreted the data, reviewed the paper for content, and reviewed and contributed to the writing of all iterations of the paper, including the final version of the manuscript. HZ and NRK participated in the early preparation of the manuscript and contributed to write the revised version of the article. All authors read and approved the final manuscript.

## References

[B1] Puleo DA, Thomas MV (2006). Implant surfaces. Dent Clin North Am.

[B2] Meyer U, Buchter A, Wiesmann HP, Joos U, Jones DB (2005). Basic reactions of osteoblasts on structured material surfaces. Eur Cell Mater.

[B3] Anselme K, Linez P, Bigerelle M, Le Maguer D, Le Maguer A, Hardouin P, Hildebrand HF, Iost A, Leroy JM (2000). The relative influence of the topography and chemistry of TiAl6V4 surfaces on osteoblastic cell behaviour. Biomaterials.

[B4] Deligianni DD, Katsala N, Ladas S, Sotiropoulou D, Amedee J, Missirlis YF (2001). Effect of surface roughness of the titanium alloy Ti-6Al-4V on human bone marrow cell response and on protein adsorption. Biomaterials.

[B5] Piconi C, Maccauro G, Muratori F, Brach del Prever E (2003). Alumina and zirconia ceramics in joint replacements. Journal of Applied Biomaterials & Biomechanics.

[B6] Christel P, Meunier A, Dorlot JM, Crolet JM, Witvoet J, Sedel L, Boutin P (1988). Biomechanical compatibility and design of ceramic implants for orthopedic surgery. Ann N Y Acad Sci.

[B7] Meyenberg KH, Luthy H, Scharer P (1995). Zirconia posts: a new all-ceramic concept for nonvital abutment teeth. J Esthet Dent.

[B8] Kakehashi Y, Luthy H, Naef R, Wohlwend A, Scharer P (1998). A new all-ceramic post and core system: clinical, technical, and in vitro results. Int J Periodontics Restorative Dent.

[B9] Tinschert J, Natt G, Mohrbotter N, Spiekermann H, Schulze KA (2007). Lifetime of alumina- and zirconia ceramics used for crown and bridge restorations. J Biomed Mater Res B Appl Biomater.

[B10] Yildirim M, Edelhoff D, Hanisch O, Spiekermann H (2000). Ceramic abutments – a new era in achieving optimal esthetics in implant dentistry. Int J Periodontics Restorative Dent.

[B11] Schliephake H, Neukam FW, Urban R (1989). Titanbelastung parenchymatöser Organe nach Insertion von Titanschraubenimplantaten. Erste Ergebnisse. Z Zahnärztl Implantol.

[B12] Tschernitschek H, Borchers L, Geurtsen W (2005). Nonalloyed titanium as a bioinert metal – a review. Quintessence Int.

[B13] Valentine-Thon E, Schiwara HW (2003). Validity of MELISA for metal sensitivity testing. Neuro Endocrinol Lett.

[B14] Yamauchi R, Morita A, Tsuji T (2000). Pacemaker dermatitis from titanium. Contact Dermatitis.

[B15] Heydecke G, Kohal R, Glaser R (1999). Optimal esthetics in single-tooth replacement with the Re-Implant system: a case report. Int J Prosthodont.

[B16] Akagawa Y, Hosokawa R, Sato Y, Kamayama K (1998). Comparison between freestanding and tooth-connected partially stabilized zirconia implants after two years' function in monkeys: a clinical and histologic study. J Prosthet Dent.

[B17] Akagawa Y, Ichikawa Y, Nikai H, Tsuru H (1993). Interface histology of unloaded and early loaded partially stabilized zirconia endosseous implant in initial bone healing. J Prosthet Dent.

[B18] Kohal RJ, Klaus G (2003). Eine vollkeramische Implantatversorgung als Einzelzahnersatz. Zahnärztl Mitt.

[B19] Rothamel D, Ferrari D, Herten M, Schwarz F, Becker J (2007). Biokompatibilität und Hartgewebsintegration einphasiger oberflächenstrukturierter Zirkoniumoxidimplantate – Eine kombinierte in-vitro- und in-vivo-Studie. Implantologie.

[B20] Pearce AI, Richards RG, Milz S, Schneider E, Pearce SG (2007). Animal models for implant biomaterial research in bone: a review. Eur Cell Mater.

[B21] Hao L, Lawrence J, Chian KS (2005). Osteoblast cell adhesion on a laser modified zirconia based bioceramic. J Mater Sci Mater Med.

[B22] Jones DB, Nolte H, Scholubbers JG, Turner E, Veltel D (1991). Biochemical signal transduction of mechanical strain in osteoblast-like cells. Biomaterials.

[B23] Martin JY, Schwartz Z, Hummert TW, Schraub DM, Simpson J, J Lankford, Dean DD, Cochran DL, Boyan BD (1995). Effect of titanium surface roughness on proliferation, differentiation, and protein synthesis of human osteoblast-like cells (MG63). J Biomed Mater Res.

[B24] Lincks J, Boyan BD, Blanchard CR, Lohmann CH, Liu Y, Cochran DL, Dean DD, Schwartz Z (1998). Response of MG63 osteoblast-like cells to titanium and titanium alloy is dependent on surface roughness and composition. Biomaterials.

[B25] Fillies T, Wiesmann HP, Sommer D, Joos U, Meyer U (2005). [Osteoblast reaction on SLA and microgrooved implant surfaces]. Mund Kiefer Gesichtschir.

[B26] Boyan BD, Batzer R, Kieswetter K, Liu Y, Cochran DL, Szmuckler-Moncler S, Dean DD, Schwartz Z (1998). Titanium surface roughness alters responsiveness of MG63 osteoblast-like cells to 1 alpha,25-(OH)2D3. J Biomed Mater Res.

[B27] Kieswetter K, Schwartz Z, Hummert TW, Cochran DL, Simpson J, Dean DD, Boyan BD (1996). Surface roughness modulates the local production of growth factors and cytokines by osteoblast-like MG-63 cells. J Biomed Mater Res.

[B28] Ko HC, Han JS, Bachle M, Jang JH, Shin SW, Kim DJ (2007). Initial osteoblast-like cell response to pure titanium and zirconia/alumina ceramics. Dent Mater.

[B29] Aldini NN, Fini M, Giavaresi G, Torricelli P, Martini L, Giardino R, Ravaglioli A, Krajewski A, Mazzocchi M, Dubini B (2002). Improvement in zirconia osseointegration by means of a biological glass coating: An in vitro and in vivo investigation. J Biomed Mater Res.

[B30] Sollazzo V, Palmieri A, Pezzetti F, Bignozzi CA, Argazzi R, Massari L, Brunelli G, Carinci F (2008). Genetic effect of zirconium oxide coating on osteoblast-like cells. J Biomed Mater Res B Appl Biomater.

[B31] Palmieri A, Pezzetti F, Brunelli G, Muzio LL, Scarano A, Scapoli L, Martinelli M, Arlotti M, Guerzoni L, Rubini C (2008). Short-period Effects of Zirconia and Titanium on Osteoblast MicroRNAs. Clin Implant Dent Relat Res.

[B32] Palmieri A, Pezzetti F, Brunelli G, Zollino I, Lo Muzio L, Martinelli M, Scapoli L, Arlotti M, Masiero E, Carinci F (2008). Zirconium oxide regulates RNA interfering of osteoblast-like cells. J Mater Sci Mater Med.

[B33] Bachle M, Butz F, Hubner U, Bakalinis E, Kohal RJ (2007). Behavior of CAL72 osteoblast-like cells cultured on zirconia ceramics with different surface topographies. Clin Oral Implants Res.

[B34] Hoffmann O, Angelov N, Gallez F, Jung RE, Weber FE (2008). The zirconia implant-bone interface: a preliminary histologic evaluation in rabbits. Int J Oral Maxillofac Implants.

[B35] Ponader S, Vairaktaris E, Heinl P, Wilmowsky CV, Rottmair A, Korner C, Singer RF, Holst S, Schlegel KA, Neukam FW (2007). Effects of topographical surface modifications of electron beam melted Ti-6Al-4V titanium on human fetal osteoblasts. J Biomed Mater Res A.

[B36] Lohmann CH, Bonewald LF, Sisk MA, Sylvia VL, Cochran DL, Dean DD, Boyan BD, Schwartz Z (2000). Maturation state determines the response of osteogenic cells to surface roughness and 1,25-dihydroxyvitamin D3. J Bone Miner Res.

[B37] Guizzardi S, Galli C, Martini D, Belletti S, Tinti A, Raspanti M, Taddei P, Ruggeri A, Scandroglio R (2004). Different titanium surface treatment influences human mandibular osteoblast response. J Periodontol.

[B38] Hao L, Lawrence J, Chian KS (2004). Effects of CO2 laser irradiation on the surface properties of magnesia-partially stabilised zirconia (MgO-PSZ) bioceramic and the subsequent improvements in human osteoblast cell adhesion. J Biomater Appl.

